# Rapid functional reorganization of the targeted contralesional hemisphere induced by one week of noninvasive closed-loop neurofeedback guides motor recovery in post-stroke patients with chronic motor impairment: a phase I trial

**DOI:** 10.1038/s43856-026-01423-x

**Published:** 2026-02-13

**Authors:** Kenichi Takasaki, Seitaro Iwama, Fumio Liu, Miho Ogura-Hiramoto, Kohei Okuyama, Michiyuki Kawakami, Katsuhiro Mizuno, Shoko Kasuga, Tomoyuki Noda, Jun Morimoto, Meigen Liu, Junichi Ushiba

**Affiliations:** 1 Graduate School of Science and Technology, Keio University, Kanagawa, Japan; 2 Department of Biosciences and Informatics, Faculty of Science and Technology, Keio University, Kanagawa, Japan; 3 Department of Rehabilitation Medicine, Keio University School of Medicine, Tokyo, Japan; 4 Department of Rehabilitation Medicine, Tokai University School of Medicine, Kanagawa, Japan; 5 Department of Brain Robot Interface, Advanced Telecommunications Research Institute International, Kyoto, Japan

**Keywords:** Phase I trials, Brain-machine interface

## Abstract

**Background:**

Post-stroke hemiplegia of the upper extremities continues to pose a significant therapeutic hurdle. Contralesional uncrossed corticospinal pathways (CST) are involved in the recovery processes.

**Methods:**

We test the safety, and preliminary efficacy of targeted upregulation of uncrossed CST excitability through self-modulation of cortical activities via noninvasive brain-machine interaction training (Registered with the University Hospital Medical Information Network: UMIN000017525). In this single-arm prospective trial, eight individuals with persistent severe post-stroke motor disability voluntarily actuated their affected shoulder using a brain-computer interface (BCI) bridging the contralesional motor cortex (M1) and an exoskeleton robot. While patients attempted to elevate the affected arm, scalp electroencephalogram (EEG) signals over the contralesional M1 were processed online to provide them with feedback on M1 excitability.

**Results:**

Here we show that the BCI reconstructs neural pathways, allowing arm elevation without any adverse effects. As evidenced by an increase in primary outcome measure (Fugl- Meyer Assessment, p < 0.05, d = 1.24), seven days of consecutive system use results in rapid, sustained, and clinically significant improvement in motor function when removed from the system and promotes contralesional M1 functional remodeling.

**Conclusions:**

This closed-loop system is safe, feasible, and a promising intervention that recruits intact neural resources to allow patients to recover upper-extremity motor abilities.

## Plain language summary

During a stroke, blood flow to the brain is blocked resulting in damage to parts of the brain. This study examines a method to support recovery of arm function in individuals with severe post-stroke paralysis. The approach aimed to enhance activity in intact brain pathways on the side opposite the side damaged by the stroke. Participants engaged in training with a brain-computer interface linked to a robotic exoskeleton. While attempting to lift their impaired arm, their brain activity was monitored and used to facilitate the movement. After seven consecutive days of training, participants demonstrated clear and sustained improvements in their ability to move the paralysed limb, without any adverse effects. These findings indicate that targeted modulation of preserved brain networks may represent a safe and promising strategy to improve upper-limb recovery in people living with long-term stroke-related disability.

## Abstract (web summary)

Takasaki et al. evaluate whether closed-loop upregulation of the contralesional motor cortex activity in the post-stroke patients promotes recovery of arm motor function. The brain-computer interface-based rehabilitation reorganizes the uncrossed corticospinal pathways in the chronic phase to improve arm movement.

## Introduction

Impairment of upper-extremity motor abilities after a stroke is highly treatment resistant, and imposes a significant burden on patients and the social economy. According to meta-analysis^1^, hemiplegic upper extremities in patients with mild to moderate motor disability can be improved by constraint-induced movement therapy, bilateral upper limb training, electromyographic (EMG) biofeedback, and robot-assisted training. These treatments aim to improve the contra- or ipsilesional corticospinal pathways but are not specific to either pathway due to the nature of behavioral training.

For patients with severe disability, whose residual motor functions limit behavioral training, repetitive mental rehearsal of affected extremities movements has been used as a motor imagery therapy. However, this open-loop strategy, which lacks feedback and standardized instruction, produces inconsistent outcomes^2^. To overcome this limitation, recently proposed closed-loop approaches using functional neuroimaging techniques, including scalp electroencephalogram (EEG), aided this therapy by monitoring the induced neural excitability to provide patients with sensory feedback in a brain state-dependent manner^3,4^. Moreover, because the closed-loop approach enables flexible allocation of neural pathways recruited during the therapy, it could be optimized by targeting intact neural pathways that are directly connected to end-effector muscles in the affected side^5^.

Maximizing the efficacy of closed-loop therapy requires precise selection of the neural structures recruited during training. If flexible selection of therapeutic targets is achieved in a noninvasive manner, the focal closed-loop training would link attempted movements with the recruitment of a specific neural activity pattern in a context-specific manner^6^. Specifically, recruiting additional neural resources connected to the affected muscles, that is promoting functional neural reorganization of contralesional activity, can lead to motor recovery in arm movement involving proximal muscles bilaterally innervated by corticomotor pathways^7^. However, there are no methods to noninvasively select which pathways to recruit during the targeted movement.

In this study, we report our experience with treating eight chronic, severe post-stroke hemiplegic patients over a 7-day period of focal brain-machine interaction training using a noninvasive closed-loop brain-computer interface (BCI) between contralesional sensorimotor activities and an exoskeleton robot^8,9^. The interventions are carried out for seven consecutive days without conventional exercise to test the safety and primary efficacy. Patients underwent training for the self-modulation of contralesional primary motor cortex (M1) population activity, supported by the closed-loop system, where somatosensory and visual stimuli are provided contingently based on the amplitude modulation of EEG signals from the targeted hemisphere. Our findings demonstrate that the intervention improved motor ability through BCI training, raising the possibility that targeted functional reorganization of the motor cortical network could have been involved in post-stroke patients with severe motor disability.

## Methods

### Study design and participants

We adopted a single-center, single-arm, open-label, prospective trial with a limited number of patients to confirm the safety and preliminary clinical efficacy of the closed-loop brain-machine intervention designed to promote contralesional M1 excitability in severe chronic poststroke hemiplegic patients^12^.

The total sample size was set as 8 based on the following rationale: the minimal detectable change in the Fugl-Meyer Assessment (FMA) in chronic poststroke hemiplegic patients is reported to be 3.2 points^13^, and we aimed to test whether the intervention effectively induces detectable FMA gains. Therefore, given that the estimated Cohen’s *d* (effect size) for robot therapy in 66 patients was 1.05 (FMA change: 3.80 ± 3.6 points), the required sample size for the paired-sample *t* test to detect this effect size in the post-evaluation comparison was estimated to be 8. The study protocol aimed to have eight patients complete the study. To account for an expected dropout rate of ~20%, a total of 10 patients were initially targeted. Ultimately, 8 patients completed the study and were included in the analysis. Originally, the study protocol included plans to conduct Study 1 and 2. However, due to the impact of COVID-19, we only conducted Study 1.

Participants were recruited from stroke outpatients at Keio University Hospital with the goal of selecting chronic stroke patients (to avoid spontaneous recovery as a confounder) with severe motor deficits in the upper limbs, especially the shoulder. The inclusion criteria were as follows: (i) first unilateral, cortical, subcortical, or mixed stroke; (ii) time from stroke onset longer than 180 days; (iii) age between 20 and 80 years; (iv) knee-mouth test score on the Stroke Impairment Assessment Set (SIAS) less than four, which represents a patient can only lift the hand to the level of the nipple^14^; (v) shoulder joint elevation range of motion of 30° or more and 120° or less without causing pain; and (vii) no severe aphasia or cognitive impairment that prevents understanding the purpose of the study. Exclusion criteria were the presence of (i) severe cognitive deficits, such as unilateral spatial neglect or aphasia, precluding training; (ii)spontaneous pain, inflammation, or impingement in the shoulder joint at the start (iii) history of dislocation, fracture, or traumatic rotator cuff injury in the shoulder joint; (vi) Patients with consciousness disorders, and (v) other severe medical conditions. Finally, eight poststroke hemiplegic patients (4 males/4 females) were recruited (see supplementary information for detailed characteristics). All patients showed no signs of higher brain dysfunction or cognitive impairment, except for one individual who exhibited very mild expressive aphasia. Comprehension was well preserved in this case, and no additional cognitive deficits were noted. The authors have obtained written consent to publish the videos from the patient.

### Safety assessment

Occupational and physical therapists (M.O. and K.O.) designed the safety assessment, and rehabilitation doctors (F.L. and M.K.) checked the intervention risks according to the ISO14971 stroke rehabilitation guidelines^15^; if necessary, risk countermeasures were applied before the intervention. The safety outcome measure was the occurrence of one of five adverse events: shoulder pain, abrasion, heat burn by electrical stimulation, ischemia, and infection. These items represent the safety concerns associated with passive movement training of the shoulder joint. The safety assessment was peer-reviewed by two rehabilitation doctors, one occupational therapist, and one physical therapist and performed by one occupational therapist and one physical therapist. Participants were queried about adverse events at each study contact.

### EEG data acquisition

Scalp EEG signals were recorded with a 128-channel Geodesic EEG system (EGI, Oregon, United States) covering the whole head. Electrode impedance was kept below 40 kΩ throughout the experiment. The EEG signals were amplified and filtered between 0.01 and 70 Hz and sampled at 1000 Hz; power noise was also removed. Participants were instructed to keep their arms and hands relaxed during recordings. The bilateral electromyogram from the deltoid muscles were monitored through the experiment to ensure that there was no involuntary increase in spasticity during training and that no voluntary muscle contractions occurred during training.

### Online EEG data processing

The excitability of the contralesional motor cortex was evaluated based on changes in the spectral power of the sensorimotor rhythm (SMR) by scalp EEG. The power attenuation of SMR is thought to reflect the excitability of populational activities in M1^16^ and is referred to as event-related desynchronization of SMR (SMR-ERD). The SMR-ERD magnitude was determined relative to EEG power in a specific frequency band, such as the alpha (8–12 Hz) or beta (12–20 Hz) band. In these frequency bands, attenuation of spectral power was observed during motor-related tasks^17^. In this study, the SMR-ERD magnitude derived from the contralesional M1 was calculated during training using the following formula:

$${SMR}-{ERD}\left(f,t\right)=\frac{R\left(f\right)-A\left(f,t\right)}{R\left(f\right)}\times 100, \%$$

where *A* is the power spectrum density of the recorded EEG at frequency *f* and time *t* regarding the onset of motor imagery, and *R* is the power spectrum density of a 2-s period from 1 s after the beginning of rest to 1 s before the end of the resting period. Moreover, we selected the individual alpha and beta frequencies for both online and offline analyses to capture individually variable responsive frequency bands. The frequency band range was set to 2 Hz in 8–20 Hz (alpha + beta), as the most significant SMR-ERD magnitude was obtained in the 2 Hz frequency band during calibration sessions^18^.

EEG data of each trial was collected to calculate SMR-ERD magnitude in the following procedure based on the previous closed-loop intervention for finger motor recovery^3,19^: (1) common averaged filter, bandpass (0.01−70 Hz), and notch (50 Hz) filtering with a fourth-order Butterworth filter, (2) segmentation for 1 s of EEG data with 90% overlap, (3) Fast Fourier transform with Hann window function, (4) power spectrum density (PSD) calculation in participant’s specific frequency bands, (5) SMR-ERD calculation.

### Intervention procedures

The intervention consisted of a two-block calibration period and a four-block training session. Each block lasted ~2 min. In total, participants underwent 30 min of intervention per day (excluding the intermission and EEG cap setup time). The interventions were carried out for seven consecutive days without conventional exercise. Clinical and neurophysiological measurements were performed 1 day before and after the intervention. A custom-made exoskeleton robot (Advanced Telecommunications Research Institute International) was attached to the paralyzed arm, and the other arm was kept relaxed throughout the intervention. The exoskeleton robot mimicked the movement performed by the occupational therapist and assisted shoulder flexion with functional electrical stimulation (FES) with the following parameters: frequency: 100 Hz, pulse width: 1 ms, intensity: motor threshold. Proprioceptive feedback was provided in the form of movement assistance during the intervention using the shoulder exoskeleton driven by a pneumatic cylinder^3,20,21^. A monitor was placed 60–90 cm in front of the participants to present visual instruction and feedback about the current brain state. The combination of the FES and robotic actuation contribute to reconstruction of the naturalistic somatosensory neural responses, and is beneficial for functional recovery with BCI^22^.

The calibration session consisted of two different parts: the first part served to identify the EEG frequency band of interest for both online and offline analyses, and, in the second part, the baseline distribution of SMR power^19^ was calculated. Each part consisted of 2 runs of 10 trials, for a total of 20 trials. In the first part, participants were asked to attempt shoulder flexion at regular intervals. Each trial started with a 5-s resting epoch, followed by a 1-s preparation epoch, and ended with a 6-s motor attempt epoch. In the second part, participants were asked to relax for 12 s duration of each trial.

**Fig. 1:**
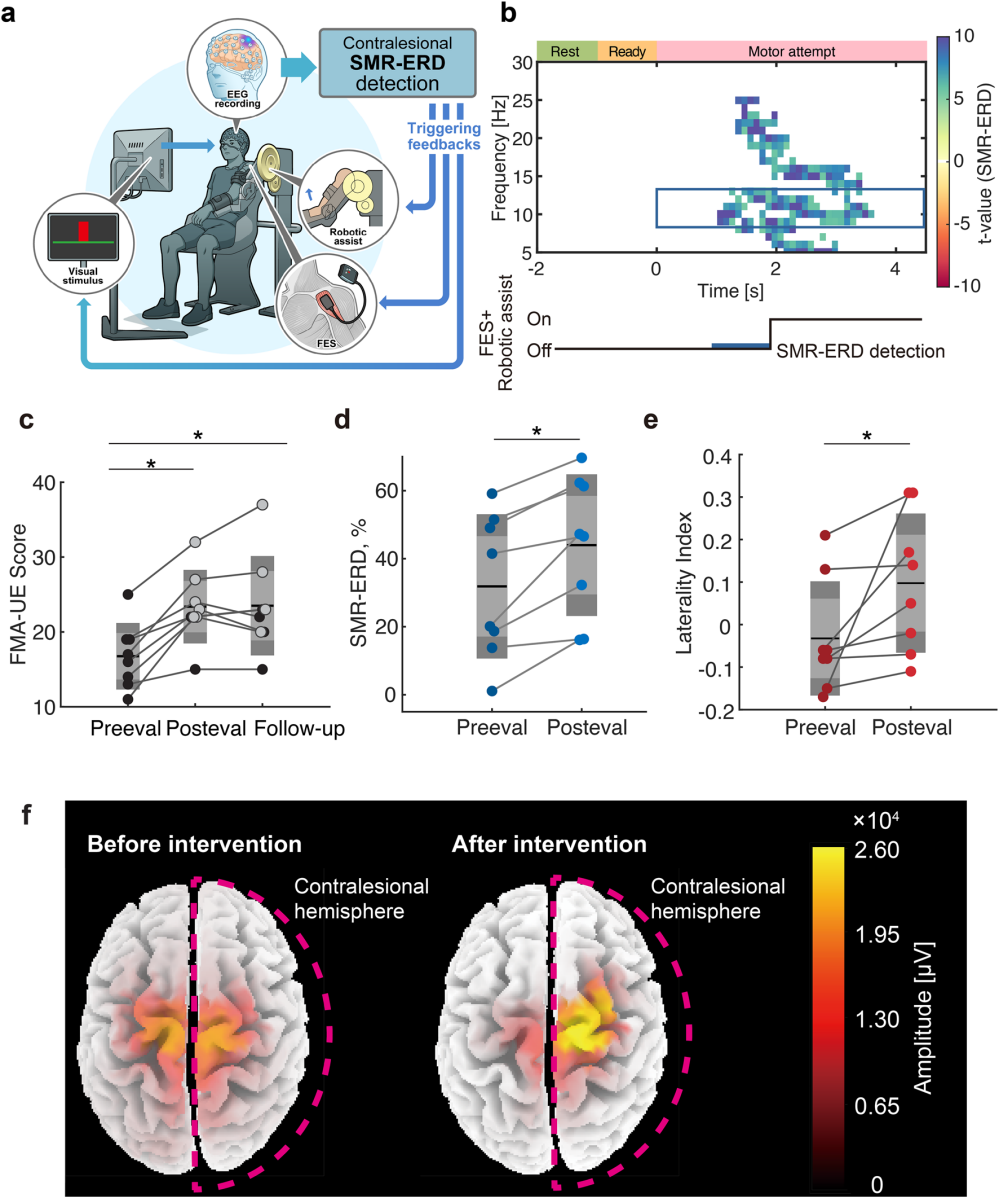
Brain-machine interaction training improves clinical scores and manipulates cortical sensorimotor representation. **a** Schematic of the intervention. When patients attempt elevation of their affected arm, contralesional sensorimotor activities are online visualized as the bar height. When continuous was observed, robotic assist and electrical stimulation reconstructed arm elevation to mimic sensory feedback. EEG: electroencephalogram; SMR-ERD: event-related desynchronization of sensorimotor rhythm; FES functional electrical stimulation. **b** Time course of the intervention and time–frequency representation of the contralesional scalp EEG data. Exoskeleton robotics assistance was initiated when SMR-ERD lasted for 1 s. **c** Changes in FMA scores (*N *= 8 *p* < 0.0001). Participants exceeding MCID were colored with light gray. FMA: Fugl-Meyer Assessment **d** Changes in SMR-ERD magnitude (*N* = 8, *p* = 0.0120). The smaller light and larger dark gray areas represent 95% confidence interval and 1 SD, respectively. The black line represents mean values. **e** Changes in SMR-ERD laterality (*N* = 8, *p* = 0.0120). **f** Whole-brain representation of SMR-ERD, estimated by cortical source estimation algorithm (see “Methods”) in the representative participant (Patient 4 in Table 1). The lateralization of SMR-ERD magnitude to the contralesional hemisphere is mainly observed around the sensorimotor cortex. One-way ANOVA with post-hoc Bonferroni-corrected *t*-test was conducted (*: *p* < 0.05).

In the training sessions, participants were asked to attempt shoulder flexion (Supplementary Fig. 1). Training consisted of 10 blocks of 10 trials, for a total of 100 trials. Each trial started with a resting period that lasted 4–9 s, followed by a 1-s preparation period and finally a 6-s MI period. The resting period finished when the SMR power was within 1 SD of the mean estimated in the calibration session. If the power did not meet the criteria, the trial ended without going to the next period. During the task period, visual SMR-ERD feedback was presented. When the participants maintained SMR-ERD over 30% for 1 s, robotic assistance with shoulder flexion and FES were triggered (Fig. 1a).

### TMS

To evaluate the effects of the intervention, changes in corticomotor reactivity were tested using transcranial magnetic stimulation (TMS). Evaluation was performed using a Magstim 200 magnetic stimulator (Magstim, Whitland, UK) with an angulated (95°) double cone coil 1 day before and after the intervention. The coil was optimally positioned to obtain MEPs in the anterior deltoid muscle with the lowest stimulus intensity, and the position was monitored with a Brainsight TMS navigation system (Rogue Research, Cardiff, UK). MEPs of the paralyzed anterior deltoid muscle were measured during shoulder flexion at 20% of maximum isometric voluntary contraction in six of eight participants who did not have contraindications to TMS. Single-pulse TMS was applied over the contralesional (i.e., ipsilateral) and ipsilesional (i.e., contralateral) M1 at 110% active motor threshold. EMG activity from the paralyzed anterior-deltoid muscle was recorded with a pair of Ag/AgCl surface electrodes (10 mm diameter) placed with centers 20 mm apart over the muscle bellies. The amplified and bandpass filtered (5–1 kHz) raw EMG signal was initially digitized at 20 kHz sampling rate, downsampled at 5 kHz, and stored for later analysis of MEP amplitudes and latencies.

### Outcome measures

The changes in FMA of upper extremity (FMA-UE) score from pre-evaluation to post-evaluation were the primary outcome measure since it represents limb impairment in terms of synergistic motor control^23^. The minimal clinically important difference (MCID) value was set to 5 based on the initial FMA score, adopting a conservative threshold. The SIAS was the secondary outcome measure for clinical testing; this standardized measure of stroke impairment consists of 22 subcategories and has excellent interrater reliability. Motor functions of the paretic upper extremity were evaluated with the knee-mouth test and the finger test and rated from 0 to 5, with 0 indicating complete paralysis and 5 indicating no paresis. The MCID was set at 1 point, indicating that a visual change occurred. Both FMA and SIAS were measured ~1 month after intervention in a follow-up test. Evaluations of upper limb motor function were conducted by blinded occupational therapists.

The secondary outcome measures of the neurophysiological assessment consisted of the following established metrics: (1) MEP size derived from the contralesional hemisphere, (2) SMR-ERD magnitude used as a biomarker of M1 activity^3^, (3) laterality of SMR-ERD^24^, and (4) resting-state functional connectivity (rsFC) of whole-head scalp EEG signals^25,26^. We performed TMS to quantify changes in reactivity in neural pathways from the M1 to the ipsilateral muscles.

### Neurophysiological analysis -scalp EEG-

To evaluate the target specificity of the intervention designed to train contralesional hemispheric modulation, the laterality index (*LI*) of the SMR-ERD magnitude was calculated using the following formula:

$${Laterality}\,{Index}\,\left({LI}\right)=\left(\frac{{cERD}-{iERD}}{{|cERD|}+{|iERD|}}\right)$$

where cERD and iERD are the values of the contra- and ipsilesional ERD, respectively.

Given that instantaneous neural oscillatory phase patterns reflect the efficacy of neuronal communication, rsFC is thought to reflect how spatially and spectrally targeted upconditioning training changes cortical network properties. To compute rsFC from scalp EEG data, the imaginary part of coherence (iCOH) has been described as a promising analysis to reveal underlying connectivity, and iCOH-based FC measures have been provided by recent research^24,27^. Accordingly, lagged coherence, which has been developed to give an improved connectivity measure compared to the iCOH, is one of the iCOH-based connectivity measures that has been used in sensor-level EEG connectivity analysis^26^. Since EEG signals measured by our high-density sensors are sensitive to volume conduction effects, we adopted iCOH as sensor-level rsFC measures, which are robust to these effects^28,29^. The iCOH was estimated from resting-state EEG data to determine the change in intrahemispheric rsFC in both ipsilesional and contralesional hemispheres and that of interhemispheric rsFC after the intervention. iCOH between EEG channels *i* and *j* was calculated with the following procedure. First, their cross-spectrum was calculated as

$${S}_{{ij}}\left(f\right)= < {x}_{i}(f){x}_{j}^{* }(f) > $$

where ${x}_{i}(f)$ and ${x}_{i}(f)$ are the corresponding Fourier transforms of signals obtained from EEG channels *i* and *j*, respectively. Then, the complex-coherence is computed as$\,{S}_{{ii}}$ ${S}_{{jj}}$

$${C}_{{ij}}=\frac{{S}_{{ij}}(f)}{\sqrt{{S}_{{ii}}(f){S}_{{jj}}(f)}}={Re}\left({C}_{{ij}}\left(f\right)\right)+{Im}\left({C}_{{ij}}\left(f\right)\right)$$

where$\,{S}_{{ii}}(f)$ and ${S}_{{jj}}(f)$ are the auto-spectrum of channels *i* and *j*. Lagged coherence is then calculated as the normalized imaginary part of coherence using the real part of coherence:

$${Lagged}\,C{OH}=\frac{{{Im}({C}_{{ij}}\left(f\right))}^{2}}{(1-{{Re}\left({C}_{{ij}}\left(f\right)\right)}^{2})}$$

To test whether relevant network connectivity significantly increased after the intervention, we created amplitude-matched surrogate data and calculated the 95th percentile as the threshold for detecting significant FC for each pair of channels. Intra- and interhemispheric network connectivity was estimated as the average lagged COH between the channels of a given hemisphere. We selected 34 channels, excluding the peripheral channels and those that detected eye movement artifacts in our connectivity analysis. The frequency of interest was set to the subject-specific frequency band, and the beta band was considered as a control condition.

### Statistical analysis

We used repeated measures one-way ANOVA with a post hoc Dunnett’s multiple comparisons test to evaluate FMA-UE scores at baseline, post-intervention (on the next day of the last intervention), and one month after the intervention.

Changes in SMR-ERD magnitude in the contralesional hemisphere and their LI values were analyzed with the Wilcoxon signed-rank test. To determine the significant FC estimated at the sensor level, resting-state EEG data were evaluated using surrogate data^30^. The distribution of surrogate data was used to estimate the threshold at the 95th percentile for detecting significant FC in each patient. Then, a one-tailed paired-sample *t* test with FDR correction was performed to determine if significant increases in iCOH were observed at the group level. For MEP data, two-sample *t*-test was applied for data from each participant. For all statistical tests, the significance threshold was set as 0.05.

### Ethics

The intervention was conducted in accordance with the Template for Intervention Description and Replication (TIDieR) guidelines^10^ and the CONSORT extension for pilot and feasibility studies^11^. The institutional ethics review board at Keio University School of Medicine approved the study (approval 20140442 and 20241023), registered with the University Hospital Medical Information Network (UMIN) (UMIN000017525). The experiment was performed after written informed consent was obtained from the participants and conducted in accordance with the Declaration of Helsinki (Trial Registry: UMIN Clinical Trials Registry; No. UMIN000017525).

## Results

### Closed-loop brain–machine interaction training

We adopted a single-center, single-arm, open-label, prospective trial design with eight patients to confirm the safety and preliminary clinical efficacy of the targeted neural reorganization approach designed to promote contralesional M1 excitability in severe chronic post-stroke hemiplegic patients, especially those with impaired shoulder mobility. From October 10, 2016, to May 10, 2017, at the Keio University Hospital, eight post-stroke patients (4 males/4 females) participated in the study with a mean age of 58.4 years (SD 11.1) and a median time from stroke onset of 29 months (range, 14–97 months) (Table 1). Recruited patients exhibited severe hemiplegia, as indexed by the average Fugl-Meyer Assessment (FMA, 16.75 ± 4.4) at the pre-intervention evaluation.

**Table 1:** Characteristics of the patients at baseline

Patient	Stroke Type	Damaged lesion	Age, y	Time since stroke, mo	FMA	SIAS KM	Proprioceptive deficit
1	Infarction	L CR	66–70	19	25	2	None
2	Hemorrhage	L TH	51–55	26	17	2	None
3	Hemorrhage	L PU	46–50	31	19	2	Mild
4	Infarction	R CR	66–70	29	19	2	None
5	Infarction	L M1	46–50	14	11	2	None
6	Hemorrhage	L TH	71–75	97	14	2	Moderate
7	Infarction	R CR	46–50	29	16	2	None
8	Infarction	R CR	61–65	34	13	2	Mild

*R* right, *L* left, *TH* thalamus, *CR* corona radiata, *PU* putamen, *M1* primary motor cortex,

*FMA* Fugl-Meyer Assessment, *SIAS KM* Stroke Impairment Assessment Set Knee-Mouth Test.

The intervention was designed to promote functional reorganization of neural pathways originating from the contralesional M1 when patients attempted to elevate the paralyzed shoulder (Fig. 1a). To this end, we employed closed-loop brain-machine interaction training that was configured to activate the robotic device, FES, and virtual object movement contingent on the contralesional sensorimotor activities monitored by scalp EEG (Supplementary Fig. 1, Supplementary Movie 1). During training, patients were asked to elevate the shoulder on the affected side and self-modulate the bar height on the screen. The visual feedback represented contralesional M1 desynchronization evaluated by changes in the spectral power of the sensorimotor rhythm (SMR) in the scalp EEG^31^. Because multimodal studies using EEG and TMS or functional magnetic resonance imaging indicate that SMR power attenuation reflects the excitability of neural populations in M1^31,32^. Thus, the SMR modulation, referred to as SMR-ERD, is thought to be the online readout of cortical excitability. To inform participants of the presence of SMR-ERD, the robotic device and FES were activated when sustained SMR-ERD was observed for more than 1 s during the motor attempt period (Fig. 1b). The exoskeleton robot mimicked the movement performed by the occupational therapist and assisted shoulder flexion with FES. The patients performed 100 trials of BCI control training per day and the performance was maintained throughout sessions (Supplementary Fig. 2, Repeated-measures ANOVA, *p* = 0.22).

### Motor recovery induced by closed-loop brain–machine interaction training

Before and after a 7-day intervention as well as 1-month follow-up period, we examined the upper limb ability. The primary outcome measure, FMA of upper extremity (FMA-UE), significantly improved after the intervention (Fig. 1c, One-way ANOVA, *p* < 0.0001, *F* = 20.854, Post-hoc paired *t*-test, pre-evaluation and post-evaluation: *t* = 5.54, *p* < 0.001, *d* = 1.24, ${{CI}}_{95}=[{{\mathrm{3.1,10.1}}}]$; pre-evaluation and follow-up: *t* = 5.65, *p* < 0.001, *d* = 1.26, ${{CI}}_{95}=[{{\mathrm{2.07,11.4}}}]$). All patients exhibited an increase in the FMA score after the intervention (6.6 ± 3.2), and six of eight patients exceeded the minimal clinically important difference (MCID) value, which was set to 5 based on the initial FMA score^33313233^. The rest of the patients exhibited smaller yet systematic improvement. Changes in the scores were driven by the section for shoulder and wrist joint assessment (Supplementary Fig. 3, Supplementary Movie 2; the shoulder elevation angle increased from ~45.2° to 155.2°). The FMA-UE score at the one-month follow-up period indicated that five patients maintained the improved motor function exceeding MCID^33^. The rest of the patients exhibited sustained improvement relative to the pre-evaluation period. In addition, muscle tone indexed by SIAS was also improved in five patients, resulting in a significant increase between baseline and after the intervention as well as between baseline and 1 month after the intervention (Dunnett multiple comparison, *p* < 0.0001). Throughout the period, no adverse events were observed. Collectively, we found the efficacy and safety of the present intervention.

### Rapid functional reorganization of the targeted contralesional hemisphere

To test whether the observed clinical improvement is accompanied by the functional reorganization of the therapeutic target, we assessed the effects of this neurophysiological treatment on the contralesional M1. An analysis of the magnitude of contralesional SMR-ERD and its laterality revealed that SMR-ERD significantly increased after the intervention (Wilcoxon signed-rank test, *p* = 0.0120, ${{CI}}_{95}=[{{\mathrm{6.2,20.2}}}]$) and lateralized to the contralesional M1 (Wilcoxon signed-rank test, *p* = 0.0120, ${{CI}}_{95}=[{{\mathrm{0.025,0.29}}}]$) (Fig. 1d, e). These results suggest that the intervention induces contralesional SMR-ERD, as intended (Fig. 1f), ultimately allowing patients to learn to self-modulate contralesional M1 excitability.

**Fig. 2:**
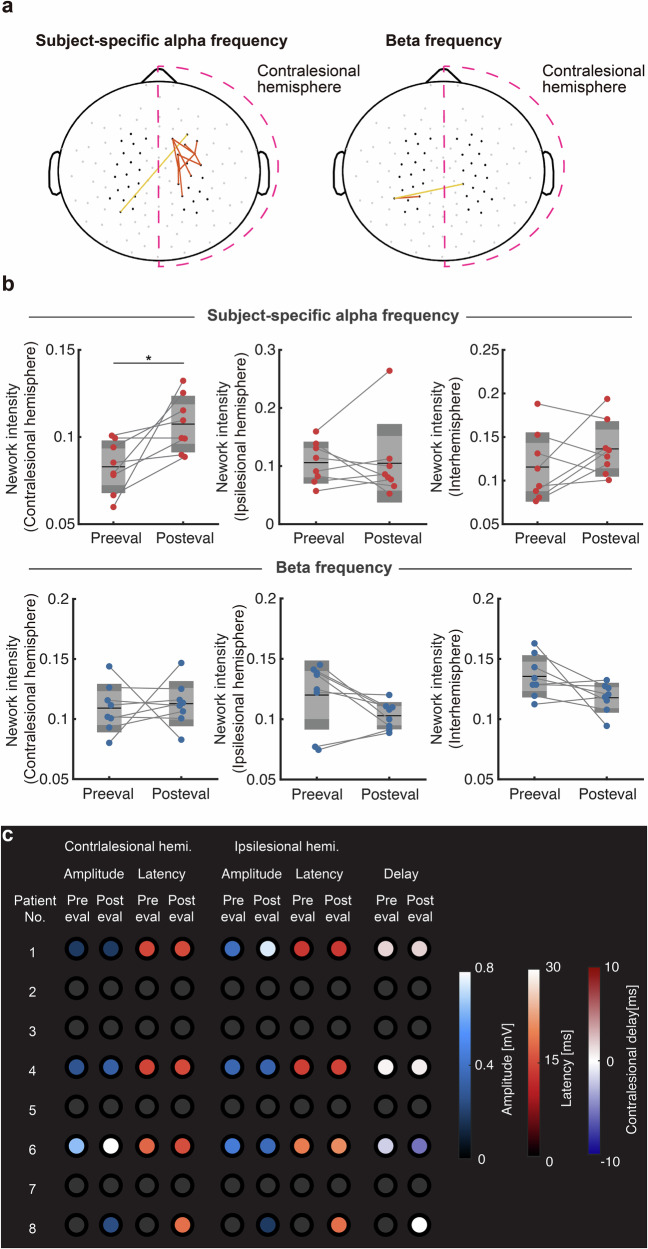
Induced sensorimotor plasticity revealed by resting-state connectivity patterns and MEP. MEP: motor-evoked potentials **a** Statistically significant connectivity patterns in subject-specific frequency in the alpha- and beta bands estimated from the resting state EEG. Orange for intrahemispheric connections, and yellow indicates interhemispheric connections. **b** Changes in the iCOH over contralesional and ipsilesional hemispheres in subject-specific alpha frequency (upper row) and beta band (lower row) for each patient (N = 8, *p* = 0.02). **c** Changes in corticomotor responsiveness assessed by TMS. Each row of data represents one patient. The magnitudes of MEPs from the contralesional and ipsilesional hemispheres are represented with a color gradient from blue to white, while latencies are represented as a color gradient from red to white. Changes in latency are indicated as the delay in the contralesional hemisphere, with negative values signifying a faster response in the contralesional corticomotor pathway than in the ipsilesional pathway. TMS transcranial magnetic stimulation.

Furthermore, we explored the whole-brain reorganization induced by the intervention. To this end, we tested the group-level changes in resting-state functional connectivity (rsFC) patterns using the imaginary part of coherency (iCOH) as a connectivity metric^14^. Significant increases in iCOH were found in the intra-hemispheric connection around the contralesional M1 (Fig. 2a, b, Paired *t*-test, *p* = 0.02, *d* = 1.02, ${{CI}}_{95}=[{{\mathrm{0.005,0.045}}}]$), within the subject-specific frequency band. The intra-hemispheric network intensity, the sum of iCOH within the contralesional hemisphere, significantly increased while no significant changes were observed in other conditions (Fig. 2b). Collectively, three scalp EEG metrics consistently indicated that functional reorganization of cortical activity is observed in the contralesional M1, where the closed-loop training was targeted.

Finally, we tested whether the cortical reorganization observed in the scalp EEG metrics also influenced the corticomotor pathways directly connected with the affected muscle. We evaluated changes in the responsiveness of the corticospinal pathway from the contralesional M1 to the anterior deltoid muscle, an agonist muscle of shoulder elevation, using motor-evoked potentials (MEPs). Corticospinal reactivity was evaluated 1 day before and 1 day after the intervention to capture intervention-induced functional plastic changes in the pathways. MEPs from the paralyzed anterior deltoid muscle were evoked using TMS during 20% of maximum isometric voluntary contraction. Six patients without TMS contraindications were tested, and data from four patients who completed the evaluation were analyzed. Contralesional MEPs, *derived from the affected anterior deltoid muscle by stimulation over contralesional hemisphere (i.e., ipsilateral to the affected muscle)*, became apparent after the intervention in three of the six tested participants (Fig. 2c, Patients 4, 6, and 8). Patient 8, who exhibited no MEP before the intervention (Supplementary Fig. 4a), demonstrated reappearance, and within-participant statistical tests revealed that MEPs derived from the contralesional hemisphere of Patients 4 and 6, and the ipsilesional hemisphere of Patient 1, exhibited significant increases (Two-sample t-test, Patient 4: *p* = 0.009, *d* = 1.07, ${{CI}}_{95}=[{{\mathrm{0.03,0.20}}}]$; Patient 6: *p* < 0.001, *d* = 3.30, ${{CI}}_{95}=[{{\mathrm{1.14,1.84}}}]$; and Patient 1*: p* < 0.001, *d* = 3.29, ${{CI}}_{95}=[{{\mathrm{0.27,0.46}}}]$, Supplementary Fig. 4b). MEP latencies were maintained after the intervention in all tested patients. Moreover, the potentiated corticomotor reactivity from the contralesional side indicated that the affected muscle is voluntarily controlled via the motor pathway from the contralesional M1. Since these metrics reflect the effects of our intervention at both cortical and corticospinal levels, these analyses allow us to interpret the neurophysiological effects of the intervention. The results of electrophysiological assessments consistently indicate that our seven-day intervention rapidly reorganized the targeted brain region.

## Discussion

Here, we aimed to demonstrate that the noninvasive digital connection between the affected muscle and the intact contralesional M1 promotes functional reorganization of the targeted cortical areas and ipsilateral uncrossed pathways. We found that 7-day consecutive use of our intervention manipulated the targeted neural pathways and led to functional motor recovery in post-stroke patients with chronic severe hemiplegia for whom it was determined that additional standard exercise would not overcome the ceiling effect. Moreover, the improvement in upper limb ability, as measured by FMA-UE scores, was greater than the MCID in six out of eight participants (FMA-UE MCID = 5)^33^. Given that impairments in motor function are treatment-resistant in patients with chronic severe hemiplegia, this single-arm, proof-of-concept study indicates the safety, and efficacy of targeted neural reorganization of the intact contralesional corticospinal pathways using closed-loop interactions with this system.

The improvement of FMA-UE was mainly driven by the shoulder joint section (section A), where our treatment targeted, and partly by the wrist joint section at the follow-up period (section B). This remote effect of functional recovery may stem from use-dependent plastic changes induced by arm joint improvement and resource allocation of the arm area to the ipsilesional side by our intervention. As a result, patients might have exhibited the functional improvement of the wrist, due to unmasking cortical areas in the ipsilesional hemisphere that could contribute to distal part recovery.

We posit that it is a promising result that six out of eight participants at the chronic stage (1–8 years from stroke onset) exceeded FMA-UE gain more than MCID, in light of the previously reported recovery trajectory, specifically that patients achieve an FMA-UE of ~20 at 30 weeks from stroke onset^34–36^. According to the data from Winters et al.^35^, all patients whose FMA scores were below 20 after 26 weeks had reached a plateau, and typically, no FMA changes were observed in this group. This observation suggests that patients in our cohort would not show any improvement if the treatment were not effective. Furthermore, data from pools with rehabilitation sessions once a week or more often, even during the chronic phase, indicated that our 1-week period of daily intervention could induce recovery equivalent to that within the 6- to 12-month period after onset^36^, ultimately shortening the recovery period. Although our single-arm study with small sample size would not allow to attribute the observed gains solely to the intervention, by comparison with historical datasets, it can be inferred that our intervention significantly induced functional recovery of chronic, severe upper limb functional impairment compared to interventions affecting the standard locus. Further research, in which a direct comparison is performed under a controlled study design, is needed to obtain proof-of-concept data.

To examine whether our intervention successfully modulated the targeted neural structures as designed, we sought the neurophysiological signatures of the functional reorganization of the contralesional M1. The contralesional SMR-ERD magnitude was the target of self-regulation during the intervention and successfully lateralized across participants. It suggests that the intervention successfully induced the hemispheric activation of the contralesional hemisphere. Related, rsFC change over the contralesional hemisphere, was mainly found in the frequency used for machine actuation during the intervention, especially for the contralesional M1 and premotor areas and their surrounding channels. Since these channels are positioned near M1 and the premotor cortex of the right hemisphere, the observed FC changes induced by our intervention might reflect the reinforced connectivity network among M1 and the premotor cortex in the contralesional hemisphere. In line with the present finding, motor recovery has been reported to be accompanied by the reorganization of the sensorimotor network^25^ and a significant increase in intrahemispheric connections in the contralesional hemisphere for patients who achieved good recovery after stroke^19^. Given that a positive contribution of the contralesional cortical network for learning novel motor skills was found in post-stroke hemiplegic patients, these network changes may underlie the successful control of excitability in the target region during the intervention and motor recovery.

In addition to the EEG signatures, we examined the small number of patients in TMS experiments to further evaluate the corticospinal reactivity. In the group, three of four tested participants showed increased ipsilateral MEPs, and one showed the reappearance of ipsilateral MEPs. Because studies have reported a positive correlation between MEPs and SMR-ERD over the contralateral and ipsilateral hemispheres in response to unilateral motor imagery^16,37^, the self-modulation training of contralesional SMR-ERD may have induced plastic changes in the contralateral M1. However, due to the limited number of patients in this pilot TMS assessment, further studies on such neurophysiological reorganization are required.

There were several limitations of this study. First, as a single-center pilot experiment with a small sample size to test the primary safety and efficacy of the intervention, it requires further validation in larger trials with long-term follow-up. Thus, the generalizability of the findings needs to be clarified. Furthermore, while the patients were blinded to targeted channel location and the training parameters, the clinicians were not blinded; this may have influenced the clinicians’ interpretation of the therapeutic response. It is also possible that the combination of repetitive electrical stimulation with the exoskeleton robot elicited motor recovery, independent of the brain-state dependent configuration. We posit that this recovery is unlikely because recruited participants had undergone standard clinical treatment and showed no significant response before enrollment in this study. Since we confirmed the safety, and preliminary efficacy of treatment under these limitations, further controlled studies would reveal the clinical significance of the brain-machine interaction therapy and neural mechanism behind the functional recovery such as the functional remodeling of contralesional M1.

In the present study, we found that closed-loop, brain-state-dependent machine actuation promoted the functional reorganization of the contralesional M1 in patients with chronic severe post-stroke hemiplegia. We posit that this intervention would be beneficial even for patients whose EMG data do not exhibit a response and whose active range of motion of the upper extremities is limited. After our intervention, these patients could subsequently experience behavioral training, such as EMG feedback^38^. Because such a sequential combination of neural intervention and motor exercise induces a cumulative therapeutic effect^38^, the digital therapeutic approach can be combined with standard clinical treatment to expand the range of treatable conditions.

## Supplementary information

Supplementary Information

Description of Additional Supplementary files

Supplementary Movie1

Supplementary Movie 2

Supplementary Data 1

## Data Availability

The anonymized physiological signal data that support the findings of this study are not publicly due to the potential risk of participant re-identification and restrictions imposed by the ethics approval and informed consent. De-identified data may be made available to qualified researchers upon reasonable request for non-commercial research purposes, subject to approval by the study investigators and completion of a data use agreement. Requests should be submitted to the corresponding author. We aim to respond within 10 business days. The data use agreement will prohibit attempts to re-identify participants, restrict onward sharing, and require acknowledgment of the original study in any resulting publications. The source data for Figs. 1c, d, e and 2b is in Supplementary Data 1.
