# Supplementary material for: Rapid functional reorganization of the targeted contralesional hemisphere induced by one week of noninvasive closed-loop neurofeedback guides motor recovery in post-stroke patients with chronic motor impairment: a phase I trial
Source: Commun Med (Lond). 2026 Feb 13;6:163. doi: 10.1038/s43856-026-01423-x (PMC13013671; doi:10.1038/s43856-026-01423-x)
Supplement: Supplementary file 1 — Supplementary Information [file 43856_2026_1423_MOESM1_ESM.pdf]

Supplementary information about ***Rapid functional reorganization of the targeted contralesional hemisphere induced by one week of noninvasive closed-loop neurofeedback guides motor recovery in post-stroke patients with chronic motor impairment: a phase I trial***

Kenichi Takasaki, Seitaro Iwama, Fumio Liu, Miho Ogura-Hiramoto, Kohei Okuyama, Michiyuki Kawakami, Katsuhiro Mizuno, Shoko Kasuga, Tomoyuki Noda, Jun Morimoto, Meigen Liu, Junichi Ushiba

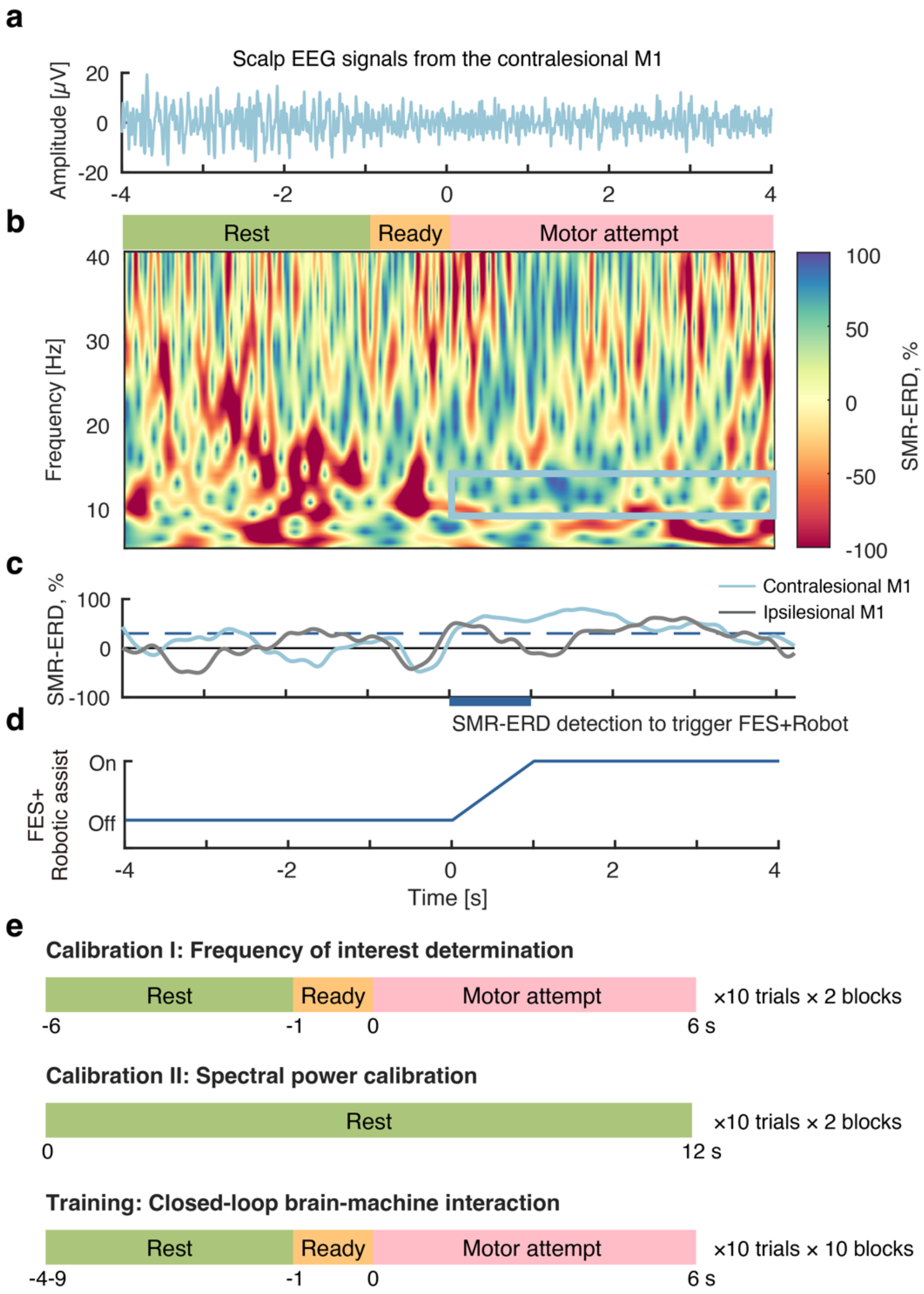

Supplementary Fig. 1 Action sequence and trial structures of the intervention procedure. **a**. raw scalp EEG signals derived from the contralesional motor cortex (M1). **b**. Time-frequency representation of EEG signals from a single trial. The blue square indicates

targeted frequency used for the SMR-ERD calculation. **c.** time course of SMR-ERD magnitude from the contra- and ipsi-lesional hemisphere. **d**. time course of electrical stimulation and robotic assist. Machines are actuated when sustained SMR-ERD magnitude was observed for one second. **e.** Trial structures in calibration and training sessions.

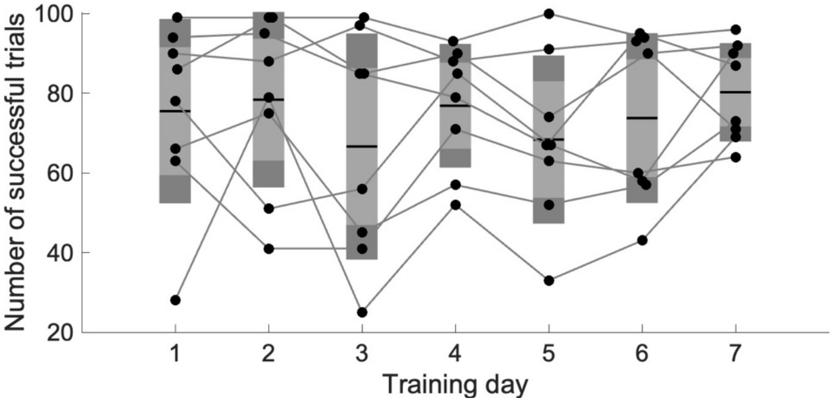

Supplementary Fig. 2 Success rate of BCI control per day. Each dot represent data from a single patient. We did not find a systematic increase at the group-level but find the maintained controllability in the patients showing motor function improvement (Repeated-measures ANOVA, p = 0.22).

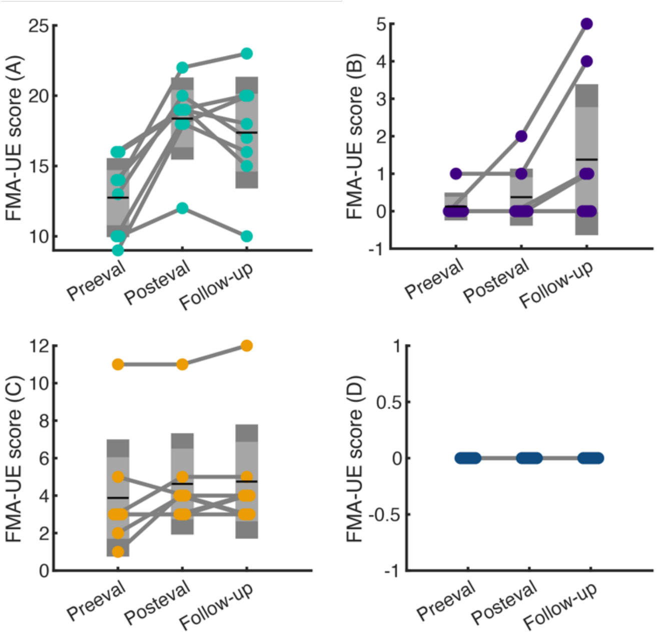

Supplementary Fig. 3 Subscores of FMA Scores. The subscores of A, B, C and D represents shoulder, wrist, hand joint function and their coordination, respectively.

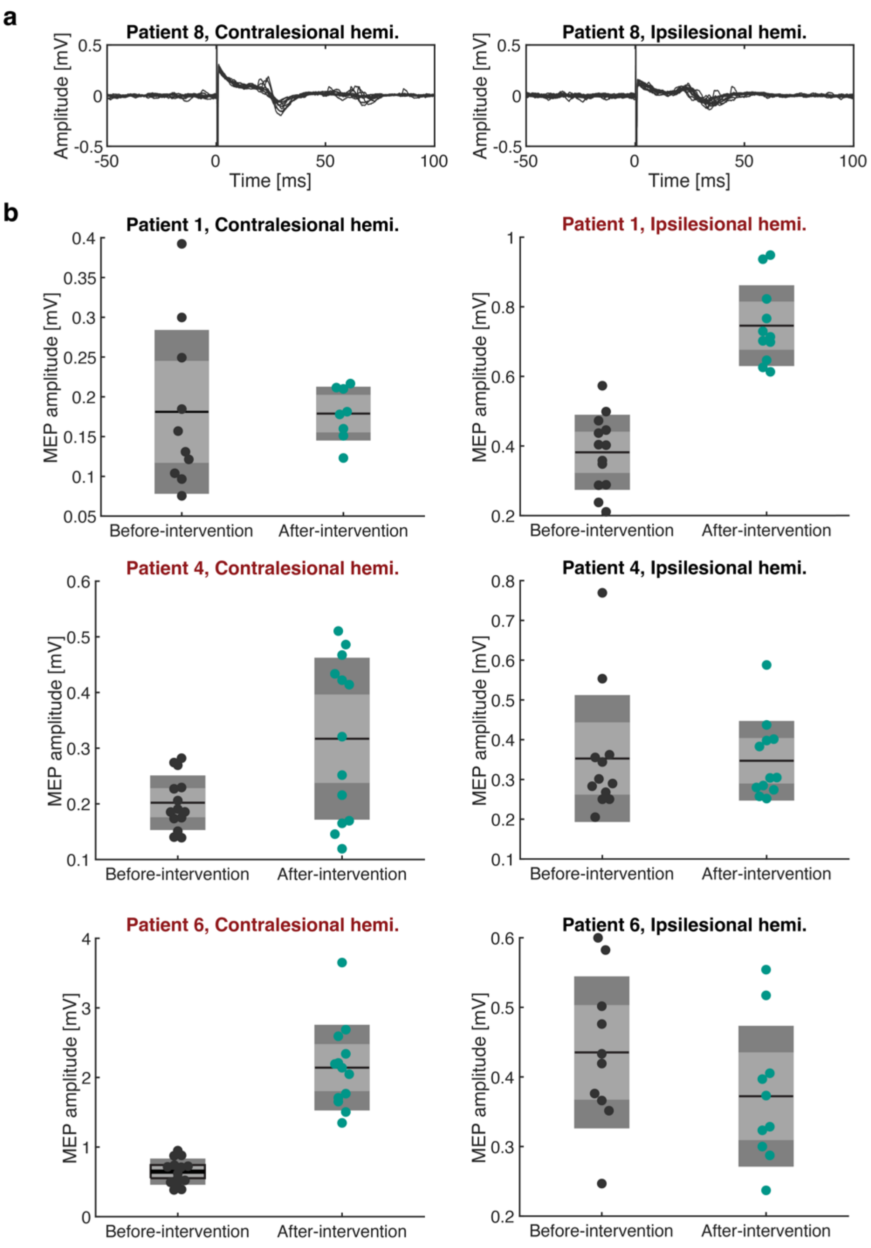

Supplementary Fig. 4 The TMS assessment reveals that functional remodeling of the corticospinal pathways was induced by the intervention. **a.** Raw signals of MEP from a representative participant at after the intervention period. Left and right panel indicate MEP from anterior deltoid muscle derived by stimulations on the contralesional and ipsilesional hemisphere, respectively. **b.** MEP magnitudes each participant. Data in each panel were subjected to two-sample t-tests with Bonferroni correction for each hemisphere. Titles whose data exhibited significant difference between before and after intervention were colored with red.

Tables

Supplementary Table 1: Averaged MEP amplitudes and latencies at pre- and post-intervention

Patient	Contrlalesional MEP [mv]		Ipsilesional MEP [mv]		Contrlalesional latency [ms]		Ipsilesional latency [ms]		contralesional delay [ms]	
	pre	post	pre	post	pre	post	pre	post	pre	post
1	0.18	0.18	0.38	0.75	15.0	15.3	13.3	13.5	1.7	1.8
2	-	-	-	-	-	-	-	-	-	-
3	-	-	-	-	-	-	-	-	-	-
4	0.27	0.32	0.35	0.34	14.6	15.2	14.1	14.4	0.5	0.8
5	-	-	-	-	-	-	-	-	-	-
6	0.65	2.14	0.44	0.37	17.6	15.6	19.5	21.1	-1.9	-5.5
7	-	-	-	-	-	-	-	-	-	-
8	N/A	0.24	N/A	0.18	N/A	18.3	N/A	18.4	N/A	-0.1

MEP: Motor evoked potential

Supplementary Table 2: FMA-UE scores

Patient	A			B			C			D			Total		
	pre	post	follow	pre	post	follow	pre	post	follow	pre	post	follow	pre	post	follow
1	14	19	20	0	2	5	11	11	12	0	0	0	25	32	37
2	16	19	18	0	0	1	3	3	4	0	0	0	19	22	23
3	10	18	20	0	0	0	1	4	3	0	0	0	11	22	23
4	14	22	23	0	0	0	3	5	5	0	0	0	17	27	28
5	16	19	15	1	1	4	2	4	3	0	0	0	19	24	22
6	13	20	17	0	0	0	3	3	3	0	0	0	16	23	20
7	10	12	10	0	0	1	3	3	4	0	0	0	13	15	15
8	9	18	16	0	0	0	5	4	4	0	0	0	14	22	20

FMA: Fugl-Meyer Assessment
