# Supplementary material for: Rapid functional reorganization of the targeted contralesional hemisphere induced by one week of noninvasive closed-loop neurofeedback guides motor recovery in post-stroke patients with chronic motor impairment: a phase I trial
Source: Commun Med (Lond). 2026 Feb 13;6:163. doi: 10.1038/s43856-026-01423-x (PMC13013671; doi:10.1038/s43856-026-01423-x)
Supplement: Supplementary file 2 — Description of Additional Supplementary files [file 43856_2026_1423_MOESM2_ESM.pdf]

Description of Additional Supplementary Files

Supplementary Movie 1- Movie of a representative trial of neurofeedback training. Authors have obtained written consent to publish the videos from the patient.

Supplementary Movie 2-Movie of a before-intervention and after-intervention range of motion. Authors have obtained written consent to publish the videos from the patient.

Supplementary Data 1- The source data for Figures 1c,1d, 1e and 2b
